# GASP1 enhances malignant phenotypes of breast cancer cells and decreases their response to paclitaxel by forming a vicious cycle with IGF1/IGF1R signaling pathway

**DOI:** 10.1038/s41419-022-05198-6

**Published:** 2022-08-30

**Authors:** Zhao Liu, Du Meng, Jianling Wang, Hongxin Cao, Peng Feng, Siyu Wu, Na Wang, Chengxue Dang, Peng Hou, Peng Xia

**Affiliations:** 1 Department of Surgical Oncology, The First Affiliated Hospital of Xi’an Jiaotong University, 710061 Xi’an, People’s Republic of China; 2 Key Laboratory for Tumor Precision Medicine of Shaanxi Province, The First Affiliated Hospital of Xi’an Jiaotong University, 710061 Xi’an, People’s Republic of China; 3 Department of Radio Oncology, The First Affiliated Hospital of Xi’an Jiaotong University, 710061 Xi’an, People’s Republic of China; 4 Department of Endocrinology, Xi’an Central Hospital, 710003 Xi’an, People’s Republic of China; 5 Department of Endocrinology, The First Affiliated Hospital of Xi’an Jiaotong University, 710061 Xi’an, People’s Republic of China

**Keywords:** Breast cancer, Oncogenes

## Abstract

There is a potential correlation between G-protein-coupled receptor-associated sorting protein 1 (GASP1) and breast tumorigenesis. However, its biological function and underlying molecular mechanism in breast cancer have not been clearly delineated. Here, we demonstrated that GASP1 was highly expressed in breast cancers, and patients harboring altered GASP1 showed a worse prognosis than those with wild-type GASP1. Functional studies showed that GASP1 knockout significantly suppressed malignant properties of breast cancer cells, such as inhibition of cell proliferation, colony formation, migration, invasion and xenograft tumor growth in nude mice as well as induction of G1-phase cell cycle arrest, and vice versa. Mechanistically, GASP1 inhibited proteasomal degradation of insulin-like growth factor 1 receptor (IGF1R) by competitively binding to IGF1R with ubiquitin E3 ligase MDM2, thereby activating its downstream signaling pathways such as NF-κB, PI3K/AKT, and MAPK/ERK pathways given their critical roles in breast tumorigenesis and progression. IGF1, in turn, stimulated GASP1 expression by activating the PI3K/AKT pathway, forming a vicious cycle propelling the malignant progression of breast cancer. Besides, we found that GASP1 knockout obviously improved the response of breast cancer cells to paclitaxel. Collectively, this study demonstrates that GASP1 enhances malignant behaviors of breast cancer cells and decreases their cellular response to paclitaxel by interacting with and stabilizing IGF1R, and suggests that it may serve as a valuable prognostic factor and potential therapeutic target in breast cancer.

## Introduction

With a top-ranked incidence and second-ranked mortality in a view of global female cancer, breast cancer is increasingly recognized as a serious and worldwide public health concern [1, 2]. Early diagnosis and comprehensive treatment have significantly improved the curing efficacy of the whole population with breast cancer, but the prognosis of patients with metastatic diseases remains poor [2]. Thus, a better understanding of the molecular mechanisms underlying the pathogenesis of breast cancer is crucial for more effective clinical treatments.

G-protein-coupled receptors (GPCRs) family is one of the biggest families of signaling receptors that regulate numerous physiological processes such as immunity, hormone signaling, nerve conduction, and cellular proliferation [3]. After binding with ligands, the activated GPCRs trigger the intracellular response cascades by catalyzing the exchange of GDP for GTP at the Gα subunit [4]. However, once exposed to agonists, GPCRs turn to rapid desensitization and internalization over time [5]. Subsequent steps of the endocytic sorting process decide whether the fate of GPCRs is recycling or degradation of GPCRs. Many intracellular G-protein-coupled receptor-binding proteins serve to regulate the cycle of GPCRs signals [6]. Among them, GASP1 has been identified as specifically targeting GPCRs to either recycling or degradation of lysosomal pathways [7, 8]. GASP1 can interact with a variety of GPCRs, such as DOR, dopamine D2, D3 receptor, the cannabinoid CB1 receptor, etc., and mediate their degradation process [9–12]. Thus, this sorting process plays a crucial role in mediating signaling cascades, mitotic growth, and cell migration. In normal settings, GASP1 is mainly expressed in neuronal cells, low or absent in other normal tissues [7]. In a pathological situation, increased expression of GASP1 has been found in brain, pancreatic, and breast cancer [13, 14]. However, its role in breast cancer has not been defined mechanistically.

In the present study, we find that GASP1 is significantly up-regulated in breast cancers, and patients with altered GASP1 have a worse prognosis than those with wild-type GASP1. Further studies reveal that GASP1 interacts with insulin-like growth factor 1 receptor (IGF1R) to prevent the MDM2-mediated ubiquitylation and degradation of IGF1R, promoting malignant phenotypes of breast cancer cells and decreasing their cellular response to paclitaxel by activating its downstream signaling pathways, such as NF-κB, PI3K/AKT, and MAPK/ERK pathways. Meanwhile, IGF1 increases GASP1 expression via a PI3K/AKT pathway-dependent manner to thereby form a vicious feedback loop propelling the progression of breast cancer and decreasing sensitivity to paclitaxel.

## Results

### Altered GASP1 status predicts poor prognosis of breast cancer patients

**Fig. 1:**
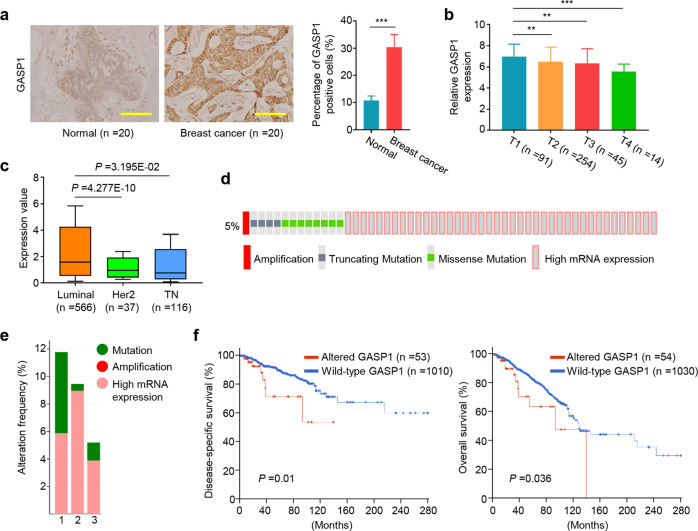
Altered GASP1 in breast cancers and its association with patient survival. **a** Immunohistochemistry was performed to determine the level of GASP1 in breast cancers and normal breast tissues (*n* = 20). Scale bar, 200 µm. **b** GASP1 expression in different stages of breast cancer patients (data from TCGA database). **c** GASP1 expression in different breast cancer subgroups, including luminal, HER2 positive, and Triple-negative types (data from UALCAN platform). **d** A total of 5% (54/1084) of breast cancers exhibit GASP1 alterations (data from cBioPortal). **e** The frequency of GASP1 alterations in different pathological types of breast cancer, including mutation, genomic amplification, and high mRNA expression (data from cBioPortal). Numbers 1, 2, and 3 represent Invasive Mixed Mucinous Carcinoma, Invasive Lobular Carcinoma, and Invasive Carcinoma (NOS, respectively. **f** The association of GASP1 alterations with disease-specific survival and overall survival in breast cancer patients. Data were presented as mean ± SD. **P < 0.01; ***P < 0.001.

We first examined GASP1 expression in 20 pairs of breast cancers and adjacent non-cancerous tissues (control subjects) by IHC assay. The results showed that GASP1 expression was significantly up-regulated in breast cancer tissues compared with control subjects (Fig. 1a). Next, we analyzed *GASP1* expression in breast cancers from The Cancer Genome Atlas (TCGA) database, and found a significant negative correlation between its expression level and tumor stage (Fig. 1b), suggesting that a high *GASP1* expression is an early event in breast tumorigenesis. Besides, we found that *GASP1* expression obviously varied in different subtypes of breast cancers via the UALCAN platform. *GASP1* expression was higher in the Luminal subtype than in HER2-positive and triple-negative subtypes (Fig. 1c).

We also investigated aberrant alterations of GASP1 in 1084 breast cancers from the cBioPortal database and found 0.09% genomic amplification (1/1084), 0.37% truncating mutations (4/1084), 0.74% missense mutations (8/1084), and 3.7% high mRNA expression (40/1084) (Fig. 1d). Given that the levels of mRNA expression are not always consistent with the protein expression, it is necessary to evaluate the significance of GASP1 protein expression in larger breast cancer patients. Moreover, there was a high proportion of these aberrant alterations in invasive breast cancers, such as invasive mixed mucinous carcinoma, invasive lobular carcinoma, and invasive carcinoma (NOS) (Fig. 1e). Further analysis indicated that patients harboring altered GASP1 had a worse disease-specific survival or overall survival than those with wild-type GASP1 (Fig. 1f). Altogether, these observations suggest potential tumor-promoting roles of GASP1 in breast cancer.

### GASP1 promotes breast cancer cell growth

**Fig. 2:**
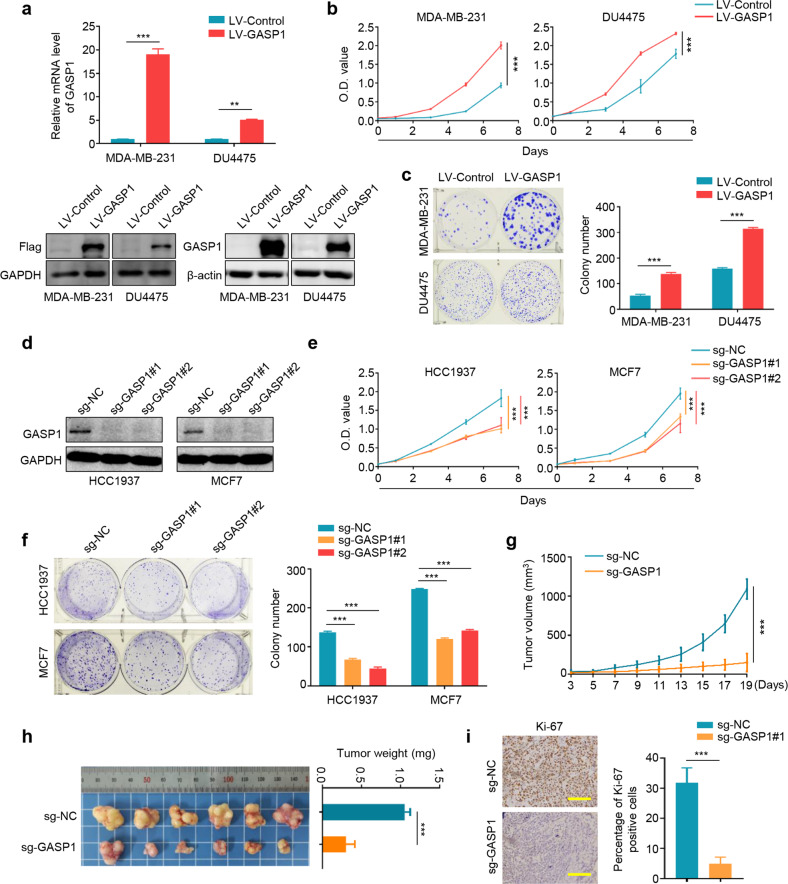
The promoting effect of GASP1 on the growth of breast cancer cells. **a** Stable expression of GASP1 in MDA-MB-231 and DU4475 cells was confirmed by qRT-PCR and western blot assays. *β-actin* was used to normalize *GASP1* expression. GAPDH and β-actin were used as the loading controls. **b** MTT showing the effect of GASP1 overexpression on the proliferation of MDA-MB-231 and DU4475 cells. **c** The effect of GASP1 overexpression on colony formation ability of MDA-MB-231 and DU4475 cells. Quantitative analysis of colony numbers is shown in the right panel. **d** GASP1 knockout by CRISPR-Cas9 technology was validated by western blot analysis. **e**, **f** GASP1 knockout in HCC1937 and MCF7 cells significantly inhibited cell growth and colony formation. **g** Xenograft tumor growth curves of GASP1 knockout MCF7 cells and control cells in nude mice (*n* = 6/group). **h** Images of the indicated xenograft tumors and statistical analysis of tumor weight. **i** The levels of Ki-67 proteins in the xenograft tumors by IHC assay (left panels). Statistical analysis of the percentage of Ki-67-positive cells was shown in the right panels. Scale bars, 200 µm. Data were presented as mean ± SD. **P < 0.01; ***P < 0.001.

To determine the biological functions of GASP1 in breast cancer, we ectopically expressed GASP1 in MDA-MB-231 and DU4475 cells by a lentivirus-mediated system (Fig. 2a) and found that GASP1 overexpression significantly promoted cell proliferation and clone formation compared with the control (Fig. 2b, c). On the other hand, we knocked out GASP1 in HCC1937 and MCF7 cells by a CRISPR/Cas9-mediated system (Fig. 2d) and found that GASP1 knockout expectedly suppressed cell proliferation and colony formation compared with the control (Fig. 2e, f).

We next established the xenograft tumor model by subcutaneously injecting GASP1 knockout MCF7 cells and control cells into mammary fat pads of nude mice, and found that GASP1 knockout significantly slowed down tumor growth (Fig. 2g) and decreased tumor weight (Fig. 2h) compared with the control. As supported, there was a lower percentage of Ki-67-positive cells in GASP1 knockout tumors than in control tumors (Fig. 2i). These results indicate oncogenic functions of GASP1 in breast cancer.

### GASP1 promotes cell cycle progression and cell migration/invasion

We determined whether altered expression of GASP1 affected cell cycle distributions of breast cancer cells. The results showed that GASP1 knockout induced the G0/G1-phase cell cycle arrest compared with the control (Supplementary Fig. 1a). Considering that cell cycle progression is tightly regulated by a series of cyclins and cyclin-dependent kinases (CDKs) [15], we thus evaluated the effect of GASP1 knockout on the expression of proteins involved in the G0/G1-phase, including Cyclin D1, cyclin E, and their relevant CDKs [16–18]. The results showed that the expression of cyclin E, CDK2, cyclin D1, and CDK4 was significantly down-regulated upon GASP1 knockout (Supplementary Fig. 1b). Conversely, ectopic expression of GASP1 up-regulated their expression (Supplementary Fig. 1c).

**Fig. 3:**
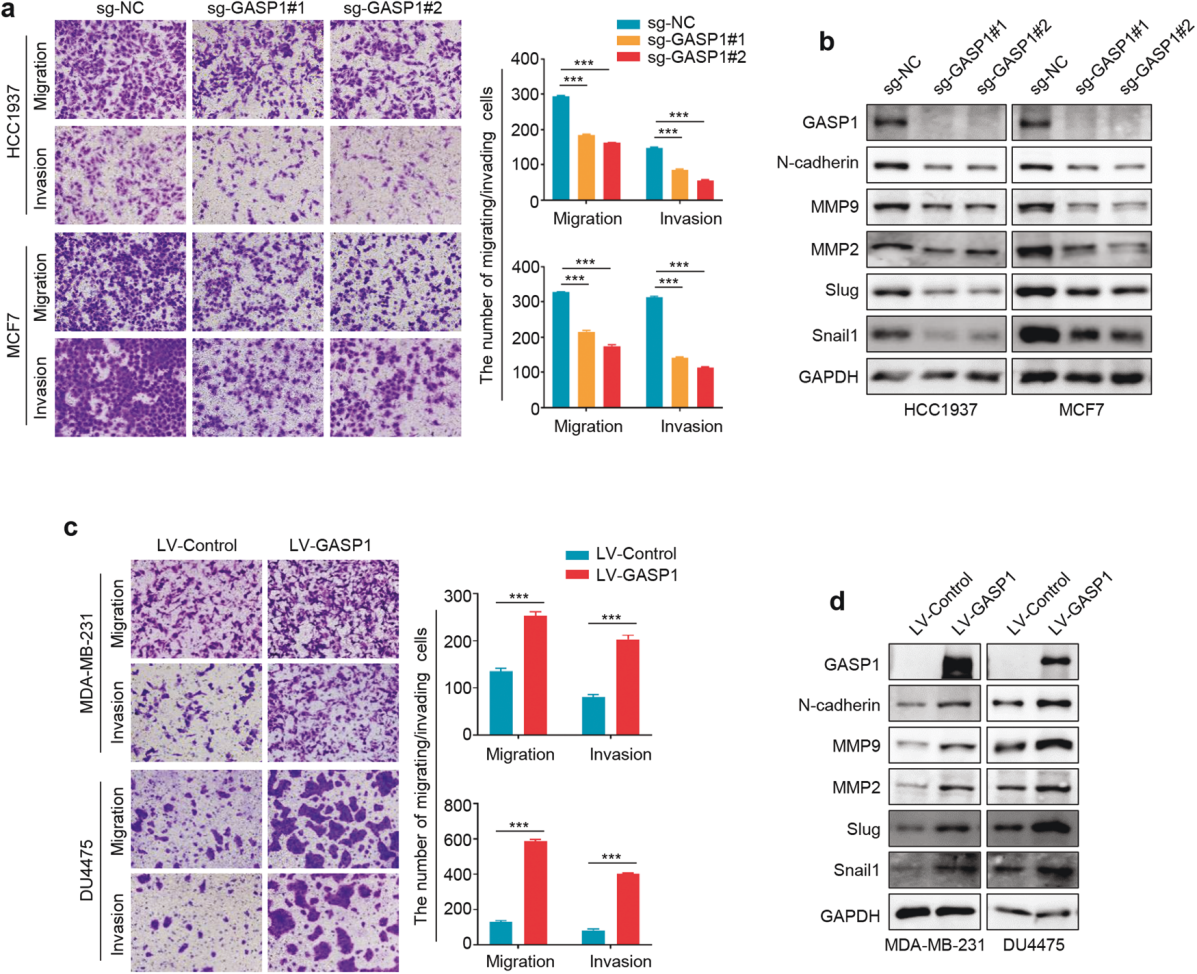
The promoting effect of GASP1 on the invasiveness of breast cancer cells. **a** GASP1 knockout suppressed the migration and invasion potential of HCC1937 and MCF7 cells. The representative pictures of migrated/invaded cells were shown in the left panels, and statistical data of cell numbers was shown in the right panels. **b** GASP1 knockout down-regulated the expression of several metastasis-related genes in HCC1937 and MCF7 cells compared with the control, including N-cadherin, MMP9, MMP2, Slug, and snail. **c** GASP1 overexpression enhanced the migration and invasion potential of MDA-MB-231 and DU4475 cells. The representative pictures were shown in the left panels and migrated/invaded cell quantification was shown in the right panels. **d** Ectopic expression of GASP1 in MDA-MB-231 and DU4475 cells up-regulated the expression of N-cadherin, MMP9, MMP2, Slug, and snail. Data were presented as mean ± SD. ***P < 0.0001.

We next studied the impact of GASP1 on the migration and invasion capability of breast cancer cells. The results showed that knocking out GASP1 in HCC1937 and MCF7 cells resulted in a suppressive effect on cell migration and invasion (Fig. 3a). Given that epithelial-mesenchymal transition (EMT) and matrix metalloproteinases (MMPs) play a vital role in the process of tumor migration and invasion [19, 20], we thus evaluated the effect of GASP1 knockout on the expression of several EMT-related genes and MMPs, and found that GASP1 depletion clearly down-regulated the expression of N-cadherin, MMP9, MMP2, Slug and Snail1 in HCC1937 and MCF7 cells compared with the control (Fig. 3b). On the contrary, ectopic expression of GASP1 in MDA-MB-231 and DU4475 cells significantly enhanced cell invasiveness, and up-regulated the expression of the above genes (Fig. 3c, d). These results indicate that GASP1 strongly correlates with metastatic phenotypes of breast cancer cells.

### GASP1 activates the IGF1/IGF1R-related signaling pathways in breast cancer cells

**Fig. 4:**
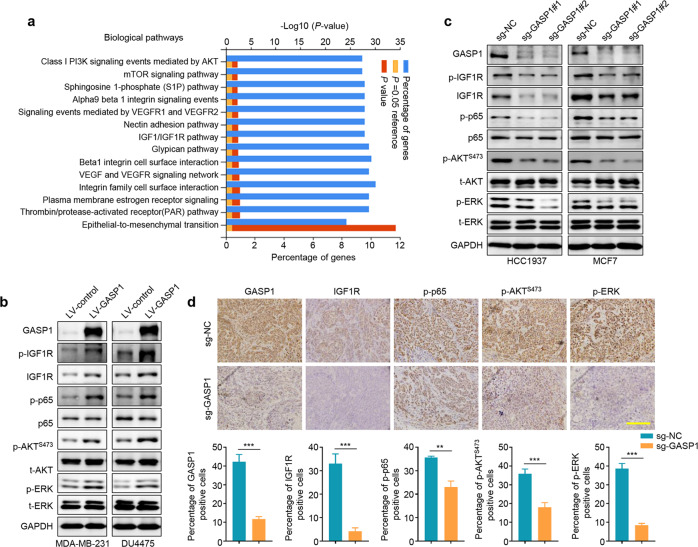
The activation of the IGF1/IGF1R-related signaling pathways by GASP1 in breast cancer cells. **a** The potential downstream pathways of GASP1 were identified by FunRich. **b** The effect of GASP1 overexpression on the activities of multiple signaling pathways, including IGF1/IGF1R1, NF-κB, PI3K/AKT, and MAPK/ERK pathways. GAPDH was used as a loading control. **c** The effect of GASP1 knockout on the activities of IGF1/IGF1R1, NF-κB, PI3K/AKT, and MAPK/ERK pathways. **d** Representative tumor sections from GASP1-knockout and control mice were subjected to IHC staining with the indicated antibodies. Scale bar, 200 µm. Data were presented as mean ± SD. **P < 0.01; ***P < 0.001.

To uncover the molecular mechanism underlying the oncogenic effects of GASP1 in breast cancer cells, we collected the data of genes co-expressed with GASP1 from the cBioportal platform and performed functional enrichment analysis using an online tool (www.funrich.org) [21]. The results showed that GASP1 was involved in regulating multiple major signaling pathways (Fig. 4a). Of them, the IGF1/IGF1R pathway and its downstream signaling pathways such as NF-κB, PI3K/AKT, and MAPK/ERK pathways have been recognized as major drivers of breast tumorigenesis and progression [22–25]. Thus, we studied whether altered expression of GASP1 modulated the activities of the IGF1/IGF1R-related signaling pathways. As shown in Fig. 4b, ectopic expression of GASP1 in MDA-MB-231 and DU4475 cells increased the levels of phosphorylated IGF1R, p65, AKT, and ERK, while virtually unchanged the levels of total p65, AKT and ERK. However, the level of total IGF1R was obviously elevated upon GASP1 overexpression. Expectedly, we observed the opposite results when GASP1 was knocked out in HCC1937 and MCF7 cells (Fig. 4c). This was also supported by the IHC staining in the GASP1 knockout and control tumors (Fig. 4d). These findings indicate that GASP1 may play its oncogenic functions in breast cancer cells by activating the IGF1/IGF1R-related signaling pathways.

### GASP1 interacts with and stabilizes IGF1R by preventing the MDM2-mediated IGF1R ubiquitination degradation

**Fig. 5:**
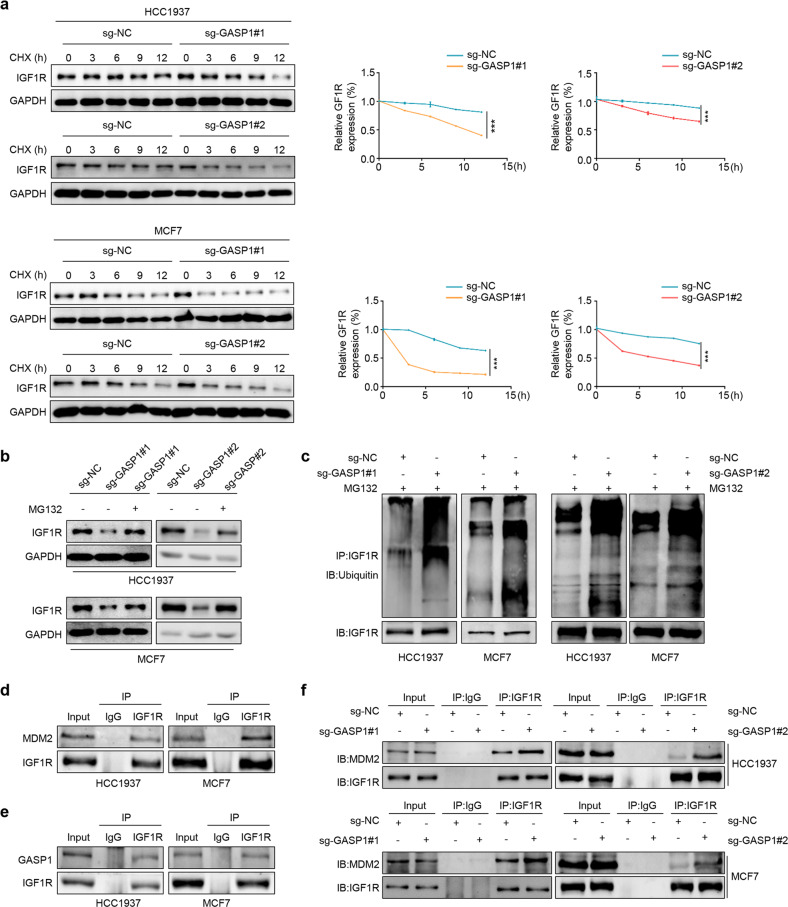
GASP1 interacts with and stabilizes IGF1R. **a** GASP1 knockout HCC1937 and MCF7 cells and control cells were treated with CHX for the indicated times. Western blot analysis was then performed to analyze the expression of IGF1R proteins (left panels). GAPDH was used as a loading control. The quantification of IGF1R proteins in the above cells was shown in the right panels. Data are presented as mean ± SD. **b** GASP1 knockout cells were treated with 20 μM MG132 for 8 h, and western blot analysis was then used to evaluate IGF1R expression. GAPDH was used as a loading control. **c** GASP1 knockout HCC1937 and MCF7 cells and control cells were treated with MG132 before harvesting. IGF1R ubiquitination was detected using immunoblot assay. **d** The interaction between MDM2 and IGF1R was detected by Co-IP assay in endogenous settings. IgG was used as a negative control. **e** The interaction between GASP1 and IGF1R was similarly detected by Co-IP assay. **f** The interaction between IGF1R and MDM2 was immunoprecipitated upon GASP1 knockout, and the expression of the indicated proteins was evaluated by immunoblotting using corresponding antibodies. ***P < 0.001.

Based on the above results, we noticed that GASP1 overexpression could up-regulate the level of total IGF1R. Thus, we speculate that it may be a major cause for GASP1 activating the IGF1/IGF1R pathway; however, what is the specific molecular mechanism? To answer this question, we first determined the effect of GASP1 on IGF1R expression at both mRNA and protein levels. The results showed that overexpression or knockout of GASP1 did not change the mRNA level of IGF1R in breast cancer cells (Supplementary Fig. 2), suggesting that GASP1 regulates IGF1R expression at the post-transcriptional level. It is clear that IGF1R stability can be regulated by MDM2-mediated ubiquitination degradation [26, 27]. To prove whether GASP1 regulates IGF1R stability, we treated breast cancer cells with protein synthesis inhibitor CHX and then performed western blot analysis to evaluate the protein expression of IGF1R upon GASP1 knockout or overexpression. The results showed that knocking out GSAP1 in HCC1937 and MCF7 cells accelerated the turnover of IGF1R proteins (Fig. 5a), while GASP1 overexpression delayed IGF1R degradation in MDA-MB-231 and DU4475 cells compared with the control (Supplementary Fig. 3). We next treated GASP1 knockout HCC1937 and MCF7 cells and their control cells with proteasome inhibitor MG132 for 8 h to block the ubiquitin-proteasome pathway and found that the inhibitory effect of GASP1 knockout on IGF1R expression could be effectively reversed by MG132 (Fig. 5b), indicating that GASP1 inhibits ubiquitination degradation of IGF1R. As supported, our data showed that GASP1 knockout elevated the ubiquitination level of IGF1R proteins in HCC1937 and MCF7 cells (Fig. 5c).

The next question is how GASP1 suppresses ubiquitination degradation of IGF1R. Considering that IGF1R can be ubiquitinated by MDM2 [26, 27], we thus speculate that GASP1 may impair the interaction between MDM2 and IGF1R, thereby protecting IGF1R from MDM2-mediated degradation. To this end, we first validated that IGF1R interacted with MDM2 and GASP1 by Co-IP assay (Fig. 5d, e). Importantly, we demonstrated that GASP1 knockout increased the interaction between MDM2 and IGF1R in both HCC1937 and MCF7 cells by reciprocal co-IP assays (Fig. 5f). These results indicate that GASP1 prevents proteasomal degradation of IGF1R by competitively binding to IGF1R with MDM2.

### GASP1 promotes breast cancer cell growth and decreases their response to paclitaxel by forming a vicious feedback loop with IGF1/IGF1R signaling pathway

**Fig. 6:**
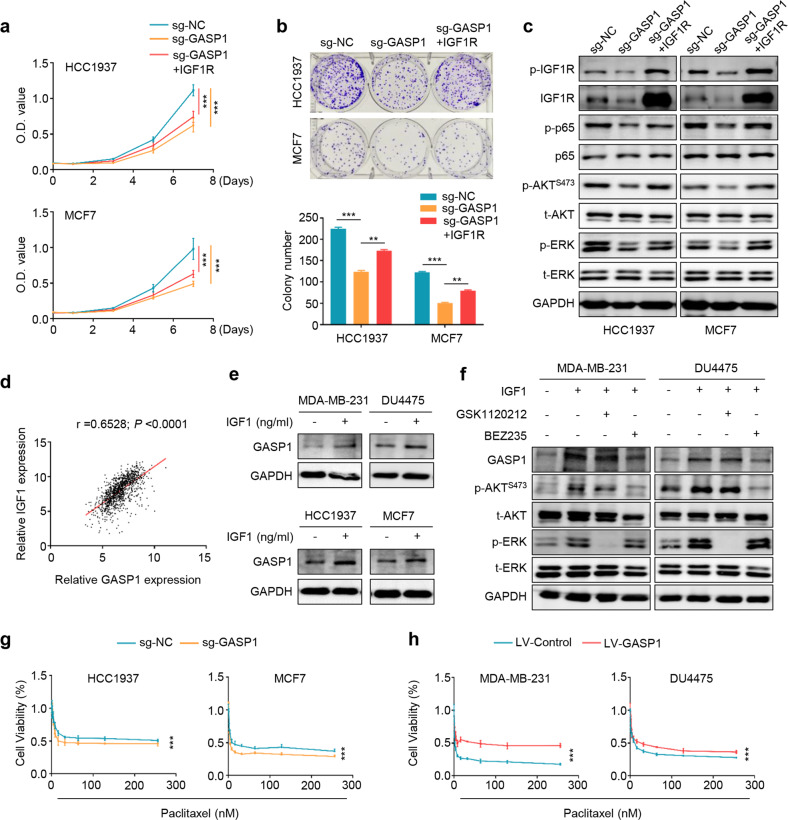
GASP1 forms a positive feedback loop with IGF1/IGF1R pathway to enhance breast cancer cell growth and decrease their response to paclitaxel. **a** The effect of ectopic expression of IGF1R in GASP1 knockout HCC1937 and MCF7 cells on cell proliferation, **b** colony formation, and the activities of IGF1/IGF1R, NF-κB, PI3K/AKT, and MAPK/ERK pathways **c**. GAPDH was used as a loading control. **d** Correlation analysis of GASP1 with IGF1 expression using the TCGA database. **e** Western blot analysis of GASP1 expression in the indicated cells treated with 100 ng/mL of IGF1 and PBS. **f** Western blot analysis of GASP1, phosphorylated AKT (p-AKT^S473^), total AKT (t-AKT), phosphorylated ERK (p-ERK), and total ERK (t-ERK) in MDA-MB-231 and DU4475 cells treated with PI3K inhibitor (BEZ235, 1 μM), MEK inhibitor (GSK1120212, 0.5 μM) and DMSO in the presence or absence of IGF1 (100 ng/mL). **g**, **h** The effect of GASP1 knockout or overexpression on the response of breast cancer cells to paclitaxel. Data were presented as mean ± SD. ***P < 0.001.

To determine IGF1R-mediated oncogenic roles of GASP1 in breast cancer cells, we ectopically expressed IGF1R in GASP1 knockout HCC1937 and MCF7 cells and found that GASP1 depletion suppressed cell proliferation compared with the control, while IGF1R overexpression could attenuate this effect (Fig. 6a). This was further supported by the results of colony formation assays (Fig. 6b). On the other hand, we knocked down IGF1R in GASP1 overexpressed MDA-MB-231 and DU4475 cells and expectedly found that GASP1 overexpression enhanced cell proliferation and colony formation, while IGF1R knockdown partially reversed these effects (Supplementary Fig. 4). In addition, ectopic expression of IGF1R could reverse the inhibitory effects of GASP1 knockout on the activities of the IGF1/IGF1R-related signaling pathways, including NF-κB, PI3K/AKT, and MAPK/ERK pathways (Fig. 6c), and vice versa (Supplementary Fig. 5).

We also found that *IGF1* expression has a significant positive correlation with *GASP1* expression by analyzing the TCGA database (Fig. 6d), suggesting that GASP1 expression may be regulated by the IGF1/IGF1R pathway. To do this, we treated breast cancer cells with exogenous IGF1 and found that IGF1 caused an elevation of GASP1 expression (Fig. 6e). Next, we treated these cells with BEZ235 (inhibitor of PI3K and mTOR) or GSK1120212 (MEK1/2 inhibitor) after pretreatment of MDA-MB-231 and DU4475 cells with exogenous IGF1. As shown in Fig. 6f, IGF1 expectedly increased the levels of phosphorylated AKT and ERK, thereby promoting GASP1 expression. BEZ235 treatment strongly inhibited AKT phosphorylation and GASP1 expression, while GSK1120212 treatment did not change GASP1 expression although ERK phosphorylation was obviously suppressed. These results suggest that GASP1 forms a positive feedback loop with the IGF1/IGF1R pathway via the PI3K/AKT pathway.

Paclitaxel is commonly used as a first-line treatment regimen for breast cancer [28, 29]. Unfortunately, dysregulation of oncogenic signaling pathways, such as NF-κB and PI3K/AKT pathways, had been widely reported to induce paclitaxel resistance [30–33]. As mentioned above, GASP1 interacted with and stabilized IGF1R, thereby activating its downstream signaling pathways such as NF-κB and PI3K/AKT pathways. Thus, we speculate that increased expression of GASP1 may decrease the response of breast cancer cells to paclitaxel by activating the IGF1/IGF1R-related pathways. To validate this, we knocked out or ectopically expressed GASP1 in breast cancer cells, and determined their effect on paclitaxel sensitivity. The results showed that GASP1 knockout enhanced the response of HCC1937 and MCF7 cells to paclitaxel (Fig. 6g), while ectopic expression of GASP1 in MDA-MB-231 and DU4475 cells decreased their response to paclitaxel (Fig. 6h), supporting the above hypothesis.

## Discussion

G-protein-coupled receptors (GPCRs) are a large family of the signal-conveying membrane in mammals [34]. Given the contribution of GPCRs in numerous physiological processes, more than 30% of clinically approved drugs are targeting this receptor family [35]. Normally, their activity is strictly regulated by multiple interacting partners that modulate their membrane targeting, intracellular trafficking, and signaling properties [5, 36, 37]. Notably, GASPs display a broad spectrum of interactions with GPCRs. In mammals, there are ten members of this family, GASP1-10 [38]. Among them, GASP1 has been reported to interact with many GPCRs C-tails [7–9, 39, 40]. A previous study found that GASP1 was highly expressed in the sera and the ductal epithelium of early-stage breast cancer patients [14]. Besides, increased expression of GASP1 was also found in brain, liver, breast, and lung cancers [13]. Recently, Professor George P. Tuszynski et al provides evidence that GASP1 may contribute to the growth of triple-negative breast cancer cells. However, its roles and underlying molecular mechanism in breast cancer have not been fully delineated.

The present study strongly supports the oncogenic roles of GASP1 in breast cancer. First, we demonstrated that GASP1 expression was elevated in breast cancers compared with control subjects, and altered GASP1 status was correlated with poor patient prognosis. Besides, we also found a negative correlation of GASP1 expression with tumor stage, suggesting that altered expression of GASP1 is an early event in breast tumorigenesis. Second, a series of functional studies determined the oncogenic functions of GASP1 in breast cancer cells, reflected by the inhibitory effects of GASP1 knockout cell proliferation, colony formation, cell migration and invasion, and cell cycle progression, and tumorigenic potential in nude mice, and vice versa. Functional enrichment analysis revealed that GASP1 may be involved in regulating the activities of the IGF1-related pathways and the procession of EMT. This was supported by our data showing that GASP1 overexpression increased the levels of phosphorylated IGF1R, p65, AKT, and ERK as well as total IGF1R, and vice versa. IGF-1 system, consisting of IGF1, IGF-binding proteins (IGFBPs), and IGF1R, acts as one of the most upstream signaling pathways to regulate various pathophysiological processes during tumorigenesis including breast cancer [41–44]. On binding to IGF1, IGF1R is phosphorylated, and subsequently activates its downstream signaling pathways, including the NF-κB, PI3K/AKT, and MAPK/ERK pathways, thereby promoting cell growth and invasiveness and suppressing cell apoptosis [44].

The present study found that GASP1 up-regulated IGF1R expression in breast cancer cells, we next explored the mechanism of GASP1-mediated IGF1R up-regulation, and speculated that GASP1 enhanced malignant behaviors of breast cancer cells by activating the IGF1/IGF1R pathway and its downstream signalings. First, we determined that overexpression or knockout of GASP1 virtually unchanged mRNA level of IGF1R in breast cancer cells, suggesting that GASP1 positively regulates IGF1R expression at a post-transcriptional level. This was supported by our data showing that GASP1 knockout accelerated IGF1R degradation and increased its ubiquitination level, and vice versa. Moreover, these effects could be effectively reversed by proteasome inhibitor MG132, further supporting the above conclusions.

Considering that MDM2 has been demonstrated to mediate IGF1R ubiquitination degradation [26, 27], we thus suppose that GASP1 may destroy the interaction between IGF1R and MDM2, as demonstrated that our data showing that GASP1 knockout caused an enhanced interaction between IGF1R and MDM2 in breast cancer cells, thereby protecting IGF1R from MDM2-mediated degradation and post-transcriptionally up-regulating its expression. Besides, we demonstrated that IGF1R-mediated oncogenic functions of GASP1 in breast cancer cells through a series of in vitro rescue experiments. These observations indicate that GASP1 promotes malignant behaviors of breast cancer cells by interacting with and stabilizing IGF1R, and subsequently activating the IGF/IGF1R pathway and its downstream signalings. Meanwhile, we also found that there was a significant positive correlation between *GASP1* expression and *IGF1* expression, and demonstrated that exogenous IGF1 significantly up-regulated GASP1 expression via the activation of the PI3K/AKT pathway. Taken together, the above results indicate that GASP1 forms a vicious self-augmenting molecular loop with the IGF1/IGF1R pathway in breast cancer cells.

Paclitaxel is widely used as a first-line chemotherapy agent for breast cancer patients [45]. However, primary or acquired resistance to paclitaxel often occurs in quite a few patients [46]. Aberrant activation of several signaling pathways has been reported to drive paclitaxel resistance, including NF-κB, PI3K/AKT, and MAPK/ERK pathways [31, 47, 48], as demonstrated by our data showing that GASP1 knockout sensitized breast cancer cells to paclitaxel, while GASP1 overexpression decreased their response to paclitaxel. These findings suggest that a vicious self-augmenting feedback loop between GASP1 and the IGF1/IGF1R-related pathways not only promotes the malignant progression of breast cancer but also contributes to the resistance of breast cancer cells to paclitaxel.

**Fig. 7:**
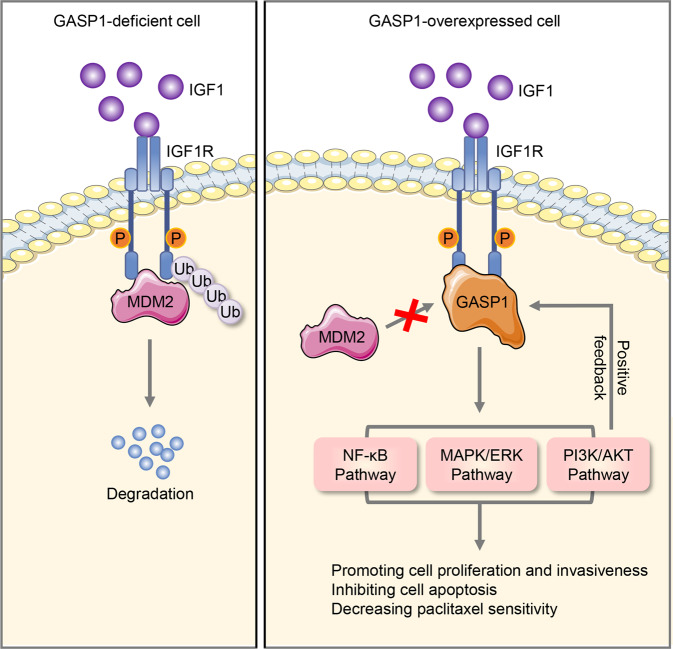
A schematic model of GASP1 promoting malignant phenotypes of breast cancer cells and decreasing their response to paclitaxel by forming a positive feedback loop with the IGF1/IGF1R pathway. In GASP1-deficient cells, IGF1R stability can be regulated by MDM2-mediated ubiquitination degradation. When GASP1 is overexpressed in breast cancer, GASP1 interacts with and stabilizes IGF1R by preventing the MDM2-mediated IGF1R ubiquitination degradation, thereby activating its downstream signaling pathways such as NF-κB, PI3K/AKT, and MAPK/ERK pathways. IGF1, in turn, stimulated GASP1 expression by activating the PI3K/AKT pathway, forming a vicious cycle propelling the malignant progression of breast cancer and decreasing the cellular response to paclitaxel in breast cancer.

In summary, the present study demonstrates that GASP1 is highly expressed in breast cancers, and plays an oncogenic role in breast cancer cells by forming a positive feedback loop with IGF1/IGF1R pathway (Fig. 7). Specifically, after binding with IGF1 and auto-phosphorylation, the activated IGF1R proteins enter the endocytosis process and are ubiquitinated by MDM2 and subsequently directed to proteasomal degradation. However, increased expression of GASP1 interacts with IGF1R and prevents IGF1R from MDM2-mediated degradation by blocking the interaction between IGF1R and MDM2, thereby activating its downstream signaling pathways, such as NF-κB, PI3K/AKT, and MAPK/ERK signaling pathways. Meanwhile, IGF/IGF1R pathway also up-regulates GASP1 expression via the activation of the PI3K/AKT pathway, forming a vicious self-augmenting feedback loop propelling the progression of breast cancer and decreasing sensitivity to paclitaxel. Thus, GASP1 may not only have a prognostic implication for breast cancer but also may be a potential therapeutic target.

## Methods

### Reagents

The proteasome inhibitor MG-132 (#S2619), eukaryote protein synthesis inhibitor CHX (#S7418), and ERK1/2 inhibitor GSK1120212 (#S2673) were purchased from Selleck Chemicals (Houston, TX, USA). Inhibitor of dual PI3K and mTOR kinase BEZ235 (#T14552) was obtained from TOP SCIENCE (shanghai, China). Recombinant human IGF-I protein (#P05019) was obtained from BioTechne (R&D Systems, MN, USA). All the reagents were used according to the manufacturer’s instructions.

### Clinical samples

A total of 20 pairs of breast cancers and adjacent non-cancerous tissues were obtained from 20 patients with infiltrating ductal breast cancer that underwent surgery at The First Affiliated Hospital of Xi’an Jiaotong University. These patients did not receive any therapeutic interventions and signed the informed consent before surgery. The histopathological type of each tissue was identified by two expert pathologists blindly and independently. This study was approved by the Institutional Review Board and Human Ethics Committee of the First Affiliated Hospital of Xi’an Jiaotong University.

### Immunohistochemistry (IHC)

The procedure was performed as described previously [49]. The information on antibodies used in this study was summarized in Supplementary Table 1.

### Human datasets

A global view of *GASP1* expression was explored in different stages and types of breast cancer tissues using the Cancer Genome Atlas (TCGA) database [50] and the UALCAN platform (http://ualcan.path. uab.edu/) [51]. Aberrant alterations of *GASP1*, including genomic amplification, mutations, and high mRNA expression, and their effect on patient survival were analyzed by using online tools (http://www.cBioPortal.org/index.do) [52, 53].

### Cell culture

Human breast cancer cell lines MDA-MB-231, DU4475, HCC1937, and MCF7 were obtained and authenticated from the American Type Culture Collection and Shanghai Bioleaf Biotech Co., Ltd. MDA-MB-231, HCC1937, and MCF7 cells were cultured in an RPMI 1640 medium supplemented with 10% fetal bovine serum (FBS), while DU4475 cells were cultured in a DMEM medium with 10% FBS at 37 °C. All cells were regularly excluded the mycoplasma contamination using the One-step Quickcolor Mycoplasma Detection Kit (Shanghai Yise Medical Technology Co., Ltd).

### Ectopic expression and knockout of GASP1

Lentivirus expressing GASP1 and control lentivirus were purchased from Shanghai Genechem Co., Ltd. One day before infection, cells were plated and allowed to grow to 30–50% confluence. Positive cells were selected by puromycin and kept in the solution containing a low concentration of puromycin for the subsequent experiments. The efficiency of overexpression was confirmed by qRT-PCR and western blot assays.

Lentivirus expressing Cas9 and sgRNAs targeting GASP1 and control lentivirus were obtained from HanBio Tech (Shanghai, China). Cells were infected with the above lentiviruses (MOI of 20) and selected with puromycin for 7 days. GASP1 expression was then determined by western blot assays. The sgRNA sequences used in this study were presented in Supplementary Table 2.

### Ectopic expression and knockdown of IGF1R

The plasmid expressing IGF1R (pLVX-AcGFP1-IGF1R) and empty vector were obtained from MiaoLingBio (Wuhan, P.R. China). Cells were plated at approximately 60%-80% confluence in a 6-well plate and transfected with 2 μg of the above plasmids with X-tremeGENE HP DNA Transfection Reagent (Invitrogen). Functional experiments were carried out 48 h after transfection.

Oligonucleotides of siRNA targeting IGF1R (si-IGF1R) and control siRNA (si-NC) were purchased from RiboBio Co., Ltd. (Guangzhou, P. R. China). Cells were transfected with a final siRNA concentration of 80 nmol/L with X-tremeGENE siRNA Transfection Reagent (Roche Diagnostics GmbH, Mannheim, Germany). The sequences of siRNA were presented in Supplementary Table 3.

### RNA extraction and quantitative RT-PCR (qRT-PCR)

RNA isolation and qRT-PCR were performed according to a previous protocol [54]. β-actin was used to normalize the expression of target genes, and primer sequences were listed in Supplementary Table 4. The relative expression level of target genes was calculated using the 2^–△△^ Ct method [55].

### Cell proliferation and colony formation assays

Cells were seeded in 96-well plates at a density of 500–1000 cells per well and incubated with 20 μL of 5 mg/mL MTT solution (Sigma-Aldrich, UK) for 4 h. Next, the medium was refreshed, and 150 μL of dimethylsulfoxide (DMSO; Sigma-Aldrich) was then added to dissolve the crystals. The O.D. values were tested on a microplate reader under ultraviolet with a wavelength of 490 nm. The experiments were performed in triplicate for each sample.

Cells are seeded in 6-well plates with an appropriate density to form clones. The culture medium was refreshed every 2–3 days. After a 2-week culture, formed clones (≥50 cells per clone) were fixed with methanol, stained with 1.25% crystal violet, and counted. Each experiment was repeated three independent times.

### Cell cycle assay

GASP1 knockout cells were harvested for 24 h and resuspended with a fresh medium. After a 48 h incubation, cells were washed with PBS, fixed in 70% ethanol for 30 min, and then stained with propidium iodide solution (50 μg/mL propidium iodide, 50 μg/mL RNase A, 0.1% Triton-X, 0.1 mM EDTA). Cell cycle distributions were evaluated by FACS analysis (BD Biosciences, San Jose, CA).

### Cell migration and invasion assays

Cell migration and invasion assays were conducted in transwell chambers (Corning Incorporated, Corning, NY, USA). The chambers were pre-coated with 15 μL of 4×dilution Matrigel (BD Biosciences, CA, USA) for cell invasion assay, while it was not necessary for cell migration assay. Cells treated with different transfections were starved overnight and then seeded in the upper chamber with 1 × 10^5^ cells in 200 μL of medium containing 0.5% FBS. Meanwhile, 750 μL of medium containing 10% FBS was added to the lower chamber. After a 24 or 72 h incubation, cells in the upper chamber were removed, and migrating or invading cells in the lower chamber were fixed with methanol for 15 min, stained with crystal violet solution, and counted under the microscope.

### Western blot analysis

Cells were lysed with RIPA buffer containing protease inhibitors on ice. Equal volumes of protein extracts were boiled for 5 min and separated by SDS-PAGE, and then transferred onto PVDF membranes (Roche Diagnostics GmbH, Mannheim, Germany). The membranes were incubated at 4 °C with primary antibodies overnight. The information of the corresponding antibodies was shown in Supplementary Table 1 On the next day, the membranes were washed five times with TBST, and incubated with HRP-conjugated secondary antibody (Thermo Fisher Scientific, CA, USA) for 1.5 h. Next, the membrane was rinsed 5 times with TBST for 25 min and then visualized by the Western Bright ECL detection system (Advansta, Menlo Park, CA, USA).

### Animal studies

Three to 4-week-old female athymic nude mice were purchased from SLAC Laboratory Animal Co., Ltd. (Shanghai, China) and raised in a specific pathogen-free (SPF) environment. To establish the tumor xenografts model, a total of 12 mice were randomly divided into two groups, and injected with ~5 × 10^6^ GASP1 knockout or control MCF7 cells mixed with Matrigel (Corning, NY, USA) into their mammary fat pads. Tumor size and body weight were measured every other day on the third day after injection. Tumor volumes were calculated by the following formula: tumor volume = length × width^2^ × 0.5. The mice were sacrificed after nearly 3 weeks and the tumor tissues were stripped and weighed for further experiments. All procedures were approved by the Institutional Animal Ethics Committee of Xi’an Jiaotong University.

### Co-immunoprecipitation (Co-IP)

After the concentration of proteins was adjusted to equal incorporation, the lysate was immunoprecipitated with respective antibodies or IgG for 4–6 h and then incubated with protein A/Gagarose beads (Catalog#: sc-2003, Santa Cruz, CA, USA) at 4 °C overnight. Next, immunoprecipitated proteins were washed with RIPA buffer and eluted from agarose beads with 5×SDS sample buffer (ZhongHuiHeCai, P.R. China). Bound proteins were then denatured and separated by western blot analysis. The information on antibodies was also presented in Supplementary Table 1.

### Statistical analysis

Student’s *t*-test and two-way ANOVA with Bonferroni posttest were conducted for data comparison of differences. The SPSS statistical package 18.0 (IBM Corp., NY, USA) was utilized to analyze statistical significance. The data were shown as mean ± standard deviation [53]. *P*-values less than 0.05 were considered statistical significance.

### Reporting summary

Further information on research design is available in the Nature Research Reporting Summary linked to this article.

## Supplementary information

Supplementary Table1

Supplementary Table2

Supplementary Table3

Supplementary Table4

Supplementary Figure legends

Supplementary Fig. 1

Supplementary Fig. 2

Supplementary Fig. 3

Supplementary Fig. 4

Supplementary Fig. 5

Reporting Summary

Full and uncropped western blots

## Data Availability

PX, PH, CD, and ZL have open access to all data and are responsible for the decision to publish this work. We declare the materials in this manuscript, including relevant data, to be freely available to any scientist wishing to use them, upon informing PX and ZL.
