# Supplementary material for: GASP1 enhances malignant phenotypes of breast cancer cells and decreases their response to paclitaxel by forming a vicious cycle with IGF1/IGF1R signaling pathway
Source: Cell Death Dis. 2022 Aug 30;13(8):751. doi: 10.1038/s41419-022-05198-6 (PMC9427794; doi:10.1038/s41419-022-05198-6)
Supplement: Supplementary file 1 — Supplementary Table1 [file 41419_2022_5198_MOESM1_ESM.docx]

**Table S1**. The antibodies used in this study

| Antibodies | Species | Catalog# | Source |
| --- | --- | --- | --- |
| Ki67 | M | 550609 | BD Pharmingen |
| GASP1(IHC) | R | 20020-1-AP | Proteintech |
| GASP1 | R | ab229627 | Abcam |
| p-IGF1R | M | sc-135767 | Santa Cruz |
| IGF1R | M | #3027 | Cell Signaling Technology |
| p-p65 | M | sc-8008 | Santa Cruz |
| p65 | M | sc-136548 | Santa Cruz |
| phospho-Akt (Ser473) | R | BS4007 | Bioworld Technology |
| total Akt | R | BS1810 | Bioworld Technology |
| phospho-Erk1/2 | R | #4370 | Cell Signaling Technology |
| total Erk | R | #4695 | Cell Signaling Technology |
| GAPDH | R | AP0063 | Bioworld Technology |
| β-actin | R | #4970 | Cell Signaling Technology |
| N-cadherin | M | sc-8424 | Santa Cruz |
| MMP9 | M | sc-21733 | Santa Cruz |
| MMP2 | R | #40994 | Cell Signaling Technology |
| Slug | R | #9585 | Cell Signaling Technology |
| Snail1 | R | #3879 | Cell Signaling Technology |
| CDK2 | M | sc-6248 | Santa Cruz |
| CDK4 | M | sc-23896 | Santa Cruz |
| Cyclin D1 | M | sc-8396 | Santa Cruz |
| Cyclin E | M | sc-247 | Santa Cruz |
| Bcl2 | M | #15071 | Cell Signaling Technology |
| Bax | M | sc-7480 | Santa Cruz |
| Flag | R | #14793 | Cell Signaling Technology |
| Ubiquitin | M | sc-8017 | Santa Cruz |
| MDM2 | M | sc-965 | Santa Cruz |
| IgG | R | ab6715 | Abcam |
| IgG | M | sc-2025 | Santa Cruz |
|  |  |  |  |

R: rabbit; M: mouse
