# Supplementary material for: GASP1 enhances malignant phenotypes of breast cancer cells and decreases their response to paclitaxel by forming a vicious cycle with IGF1/IGF1R signaling pathway
Source: Cell Death Dis. 2022 Aug 30;13(8):751. doi: 10.1038/s41419-022-05198-6 (PMC9427794; doi:10.1038/s41419-022-05198-6)
Supplement: Supplementary file 2 — Supplementary Table2 [file 41419_2022_5198_MOESM2_ESM.docx]

**Table S2**. The sgRNA sequences targeting GASP1

| **Guide RNAs** | **5’-3’ targeting sequence** |
| --- | --- |
| sgRNA1 | tgagagctttcctagaagga |
| sgRNA2 | tggtgtcagatgcagattta |
