# Supplementary material for: GASP1 enhances malignant phenotypes of breast cancer cells and decreases their response to paclitaxel by forming a vicious cycle with IGF1/IGF1R signaling pathway
Source: Cell Death Dis. 2022 Aug 30;13(8):751. doi: 10.1038/s41419-022-05198-6 (PMC9427794; doi:10.1038/s41419-022-05198-6)
Supplement: Supplementary file 3 — Supplementary Table3 [file 41419_2022_5198_MOESM3_ESM.docx]

**Table S3**. The siRNA sequences used in this study

| **siRNAs** | **Sequence (5’-3’)** |
| --- | --- |
| si-IGF1R | CGAAGATTTCACAGTCAAA |
| si-NC | CGAAGATTTCACAGTCAAA |
