# Supplementary material for: GASP1 enhances malignant phenotypes of breast cancer cells and decreases their response to paclitaxel by forming a vicious cycle with IGF1/IGF1R signaling pathway
Source: Cell Death Dis. 2022 Aug 30;13(8):751. doi: 10.1038/s41419-022-05198-6 (PMC9427794; doi:10.1038/s41419-022-05198-6)
Supplement: Supplementary file 4 — Supplementary Table4 [file 41419_2022_5198_MOESM4_ESM.docx]

**Table S4**. The primers used in this study

| **Genes** | **Forward primer (5’-3’)** | **Reverse primer (5’-3’)** |
| --- | --- | --- |
| *GASP1* | AGGCCAAGGCAATACCTGT | TGCTTGGTAATGGGCCTTCC |
| *IGF1R* | CCTGCACAACTCCATCTTCGTG | CGGTGATGTTGTAGGTGTCTGC |
| *β-actin* | GCACAGAGCCTCGCCTT | GTTGTCGACGACGAGCG |
