# Supplementary material for: GASP1 enhances malignant phenotypes of breast cancer cells and decreases their response to paclitaxel by forming a vicious cycle with IGF1/IGF1R signaling pathway
Source: Cell Death Dis. 2022 Aug 30;13(8):751. doi: 10.1038/s41419-022-05198-6 (PMC9427794; doi:10.1038/s41419-022-05198-6)
Supplement: Supplementary file 5 — Supplementary Figure legends [file 41419_2022_5198_MOESM5_ESM.docx]

**Figure legends**

**Supplementary Fig. 1 GASP1 knockout induces G0/G1 cell cycle arrest in breast cancer cells.** **a** The distribution of cell cycle was analyzed in GASP1 knockout HCC1937 and MCF7 cells and control cells by flow cytometry. **b**, **c** The effects of GASP1 knockout and overexpression on the expression of Cyclin E, Cyclin D1, CDK4, and CDK2 were determined by western blot analysis. GAPDH was used as a loading control. **, *P* < 0.01; ***, *P* < 0.001.

**Supplementary Fig. 2 GASP1 alterations fail to change the mRNA level of IGF1R.**

**a**, **b** GASP1 overexpression and GASP1 knockout fail to change the mRNA level of IGF1R in the indicated breast cancer cells by qRT-PCR. Data were presented as mean ± SD. ***, *P* < 0.001.

**Supplementary Fig. 3 GASP1 increases the stability of IGF1R proteins.** A cycloheximide chase was performed to determine IGF1R biogenesis. GASP1 overexpression cells were treated with CHX for the indicated times (left panels). IGF1R expression was then measured by western blot analysis. GAPDH was a loading control. Quantitative analysis of IGF1R protein expression was shown in the right panels. Data were presented as mean ± SD. ***, *P* < 0.001.

**Supplementary Fig. 4 IGF1R knockdown decreases the promoting effects of GASP1 overexpression on cell proliferation and colony formation. a** The effect of IGF1R knockdown in MDA-MB-231 and DU4475 cells stably expressing GASP1 on cell proliferation and **b** colony formation. **, *P* < 0.01; ***, *P* < 0.001.

**Supplementary Fig. 5 IGF1R knockdown partially reverses the promoting effects of GASP1 overexpression on IGF1/IGF1R-related signaling pathways.**

IGF1R knockdown attenuates the promoting effect of GASP1 overexpression on the activities of IGF1/IGF1R, NF-κB, PI3K/AKT, and MAPK/ERK signaling pathways. GAPDH was used as a loading control.
