# Supplementary figures and images for: GASP1 enhances malignant phenotypes of breast cancer cells and decreases their response to paclitaxel by forming a vicious cycle with IGF1/IGF1R signaling pathway
Source: Cell Death Dis. 2022 Aug 30;13(8):751. doi: 10.1038/s41419-022-05198-6 (PMC9427794; doi:10.1038/s41419-022-05198-6)

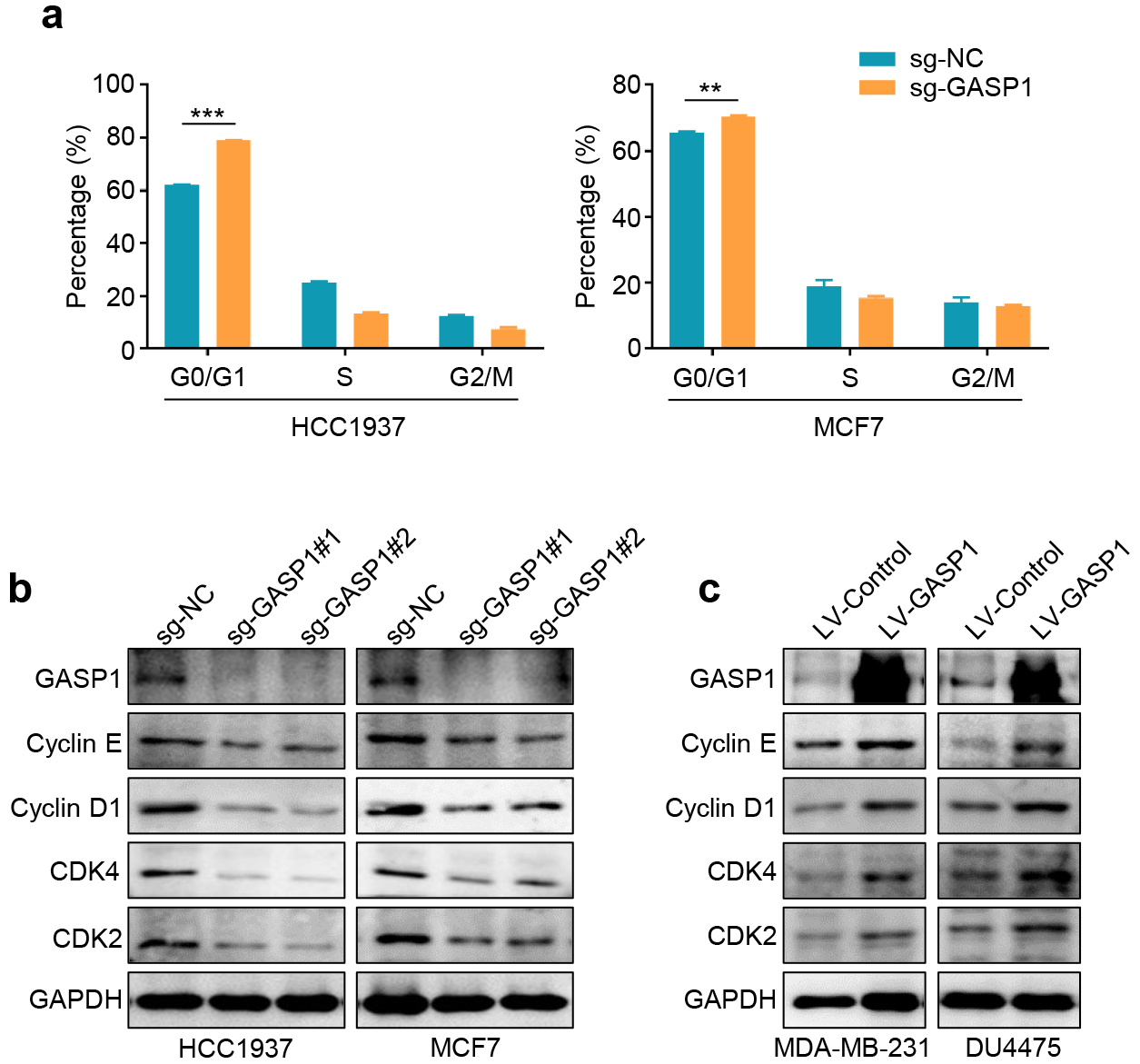

Supplement: Supplementary file 6 — Supplementary Fig. 1 [file 41419_2022_5198_MOESM6_ESM.tif]

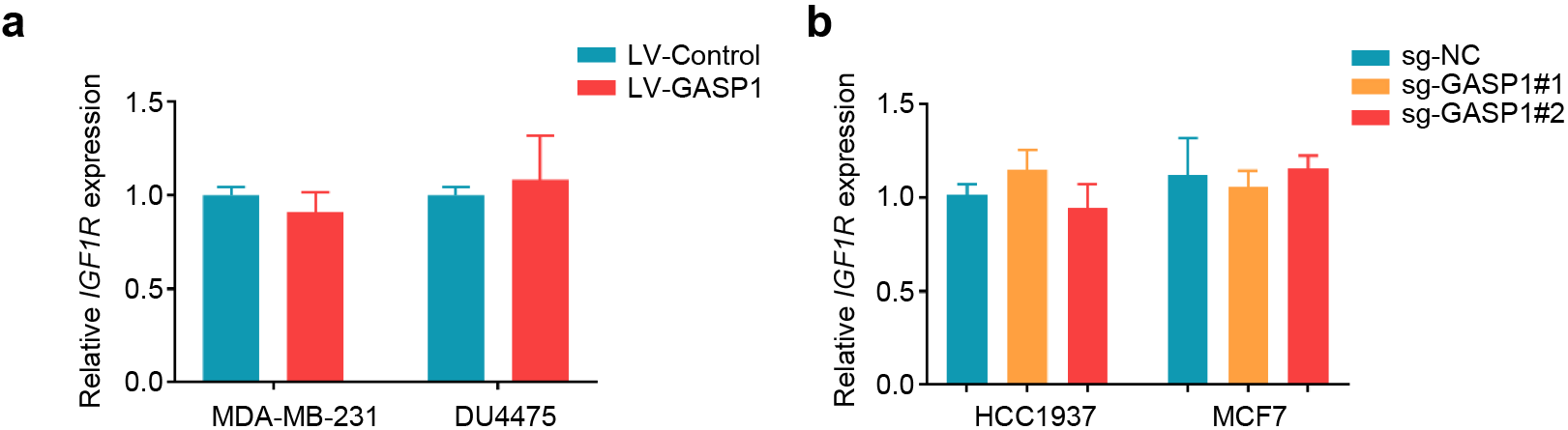

Supplement: Supplementary file 7 — Supplementary Fig. 2 [file 41419_2022_5198_MOESM7_ESM.tif]

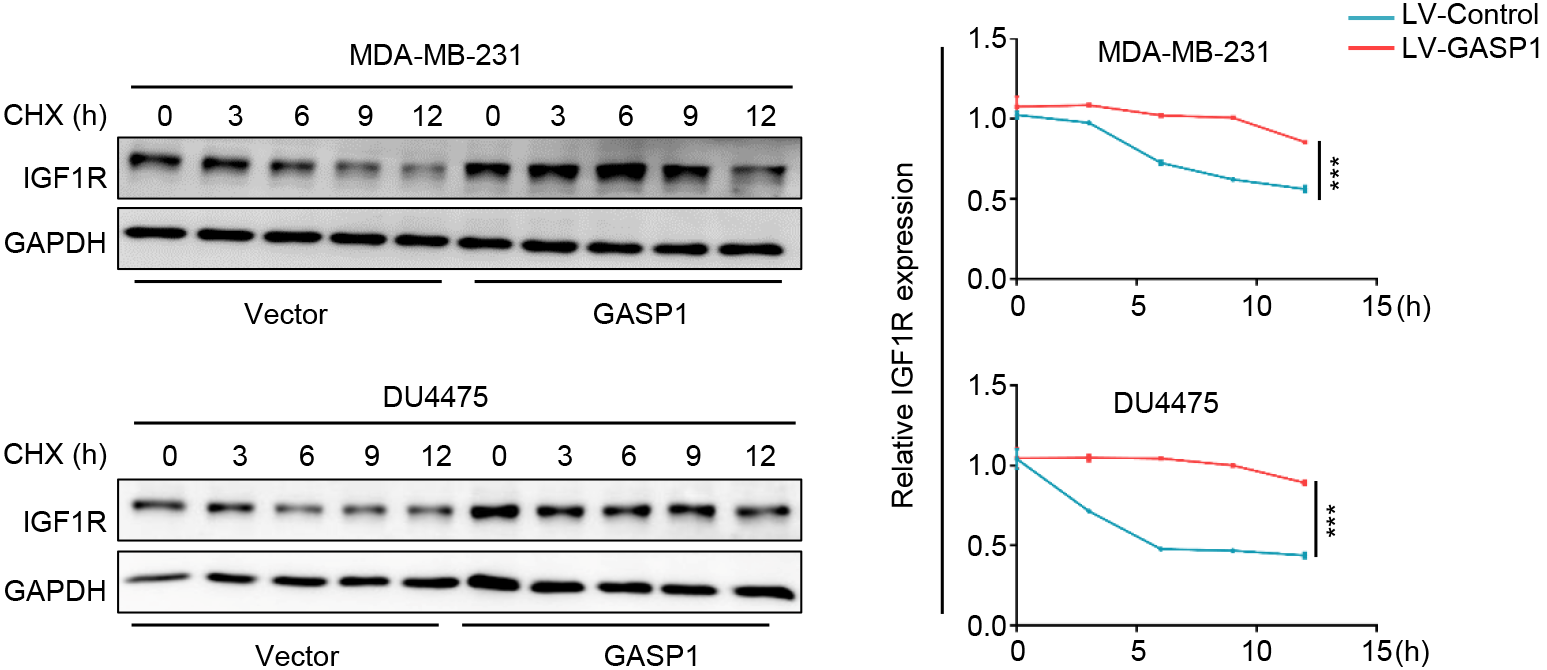

Supplement: Supplementary file 8 — Supplementary Fig. 3 [file 41419_2022_5198_MOESM8_ESM.tif]

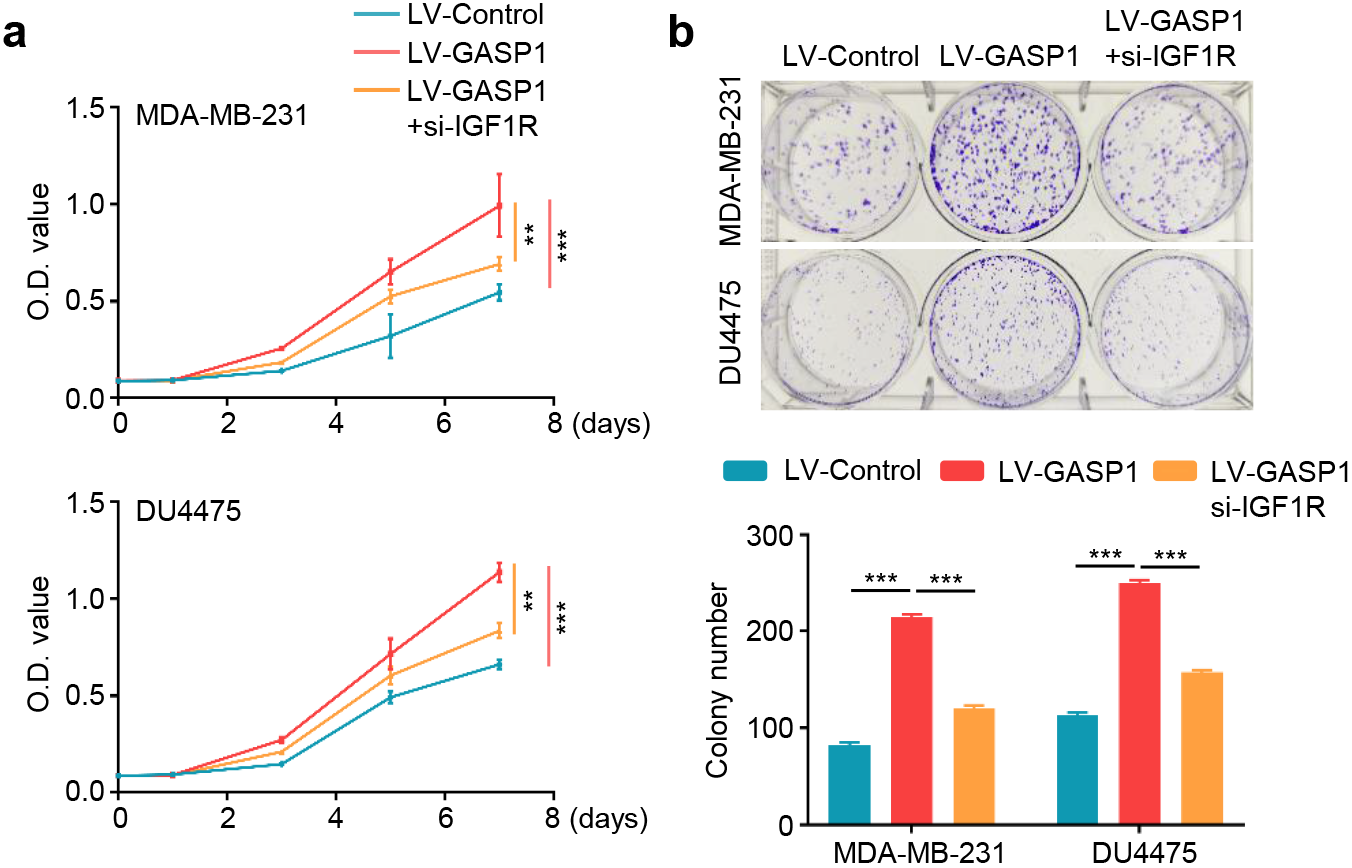

Supplement: Supplementary file 9 — Supplementary Fig. 4 [file 41419_2022_5198_MOESM9_ESM.tif]

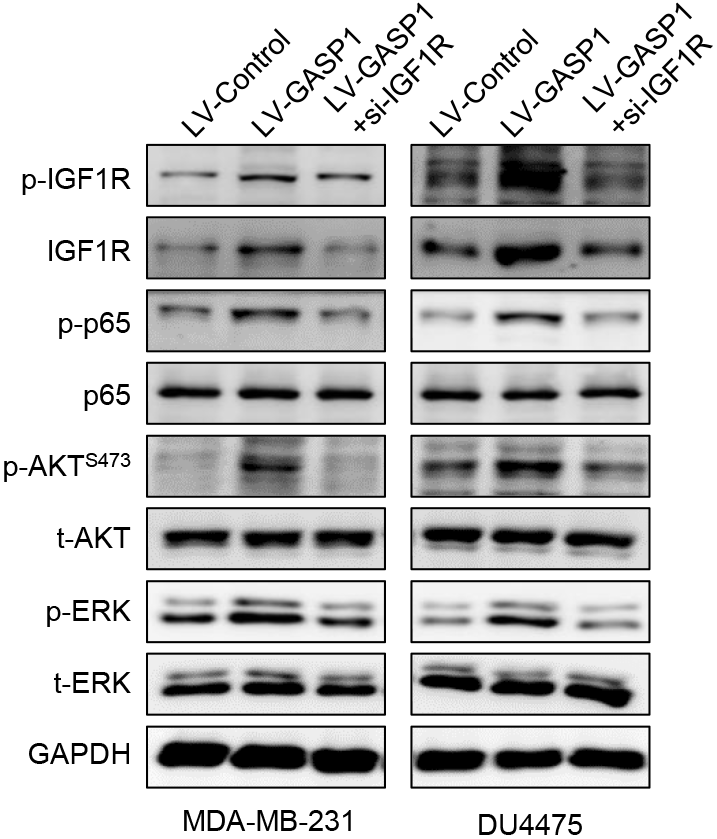

Supplement: Supplementary file 10 — Supplementary Fig. 5 [file 41419_2022_5198_MOESM10_ESM.tif]

**a**

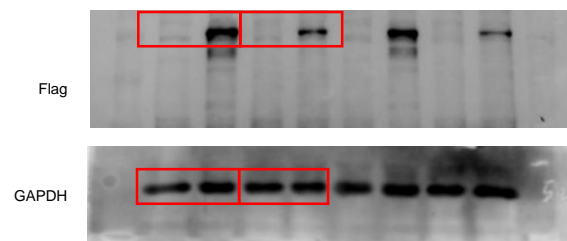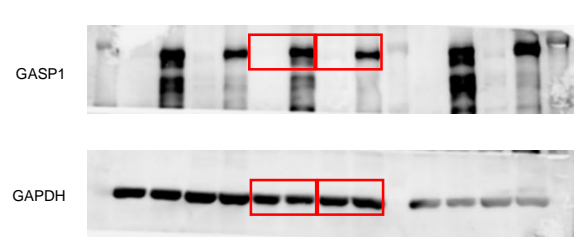

**d**

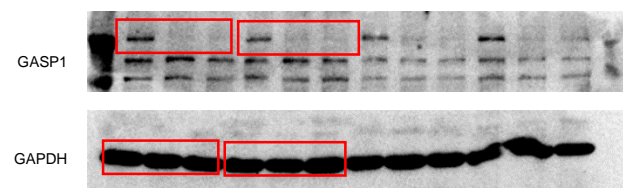

**c**

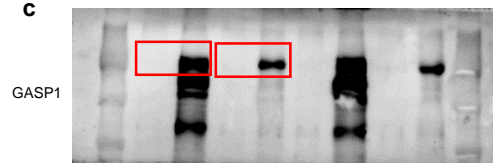

N-cadherin

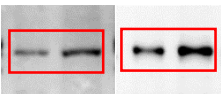

MMP9

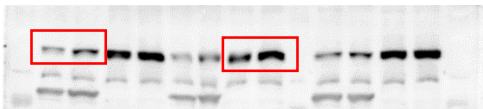

MMP2

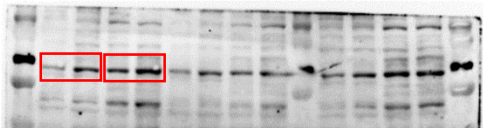

Slug

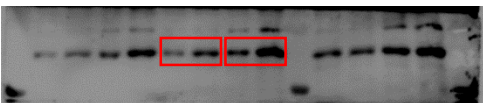

Snail1

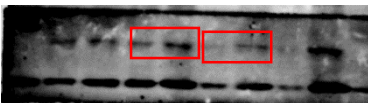

GAPDH

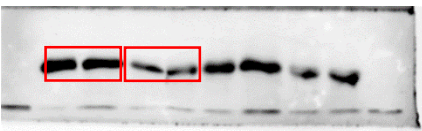

**d**

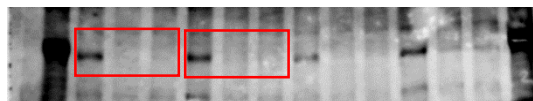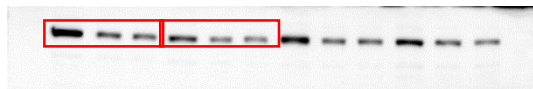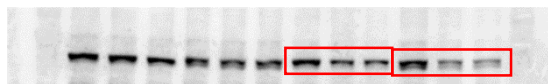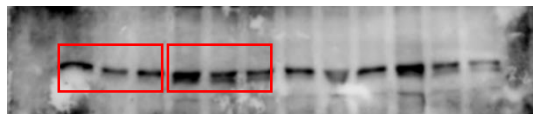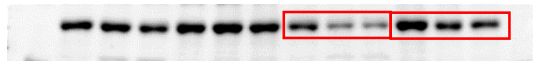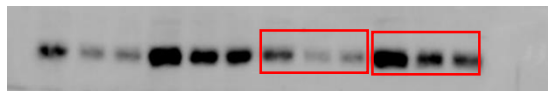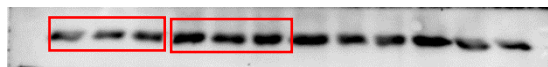

**b**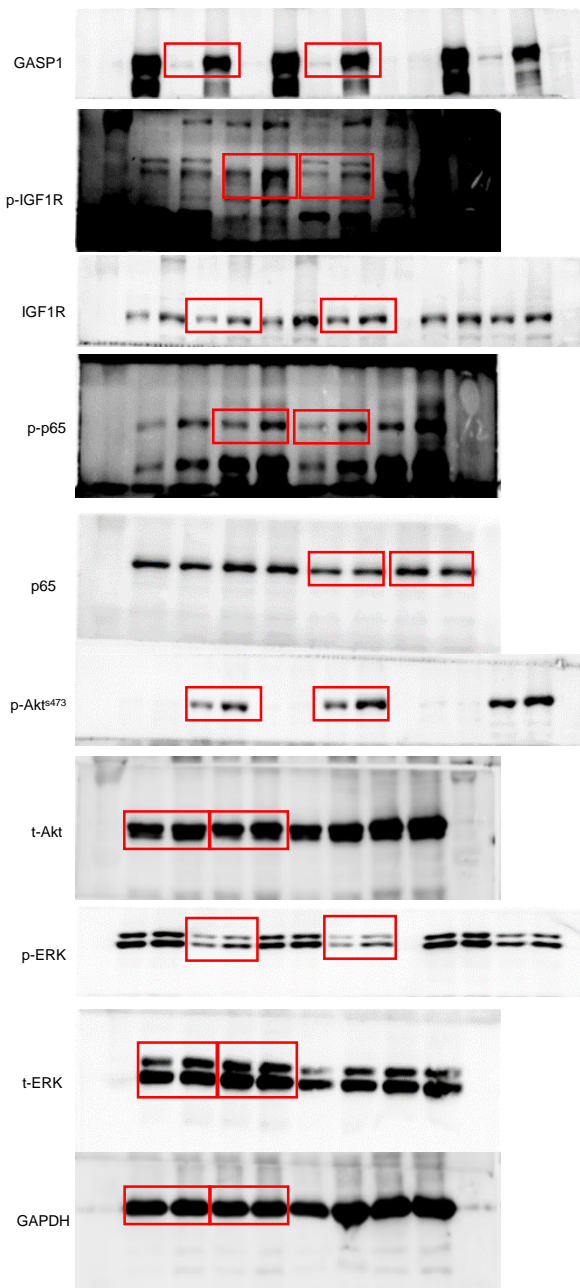**c**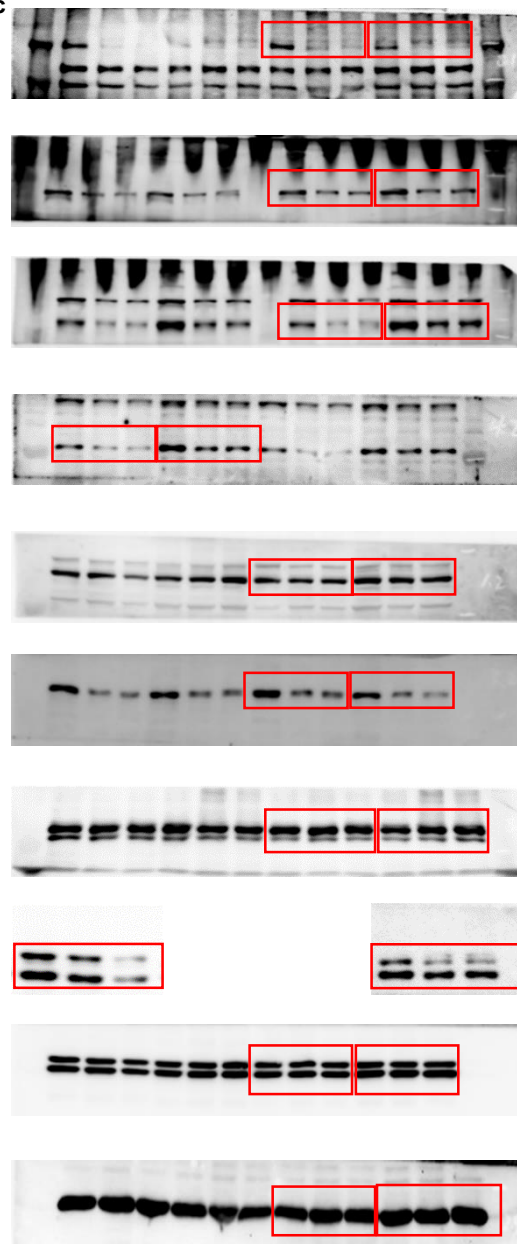

**a**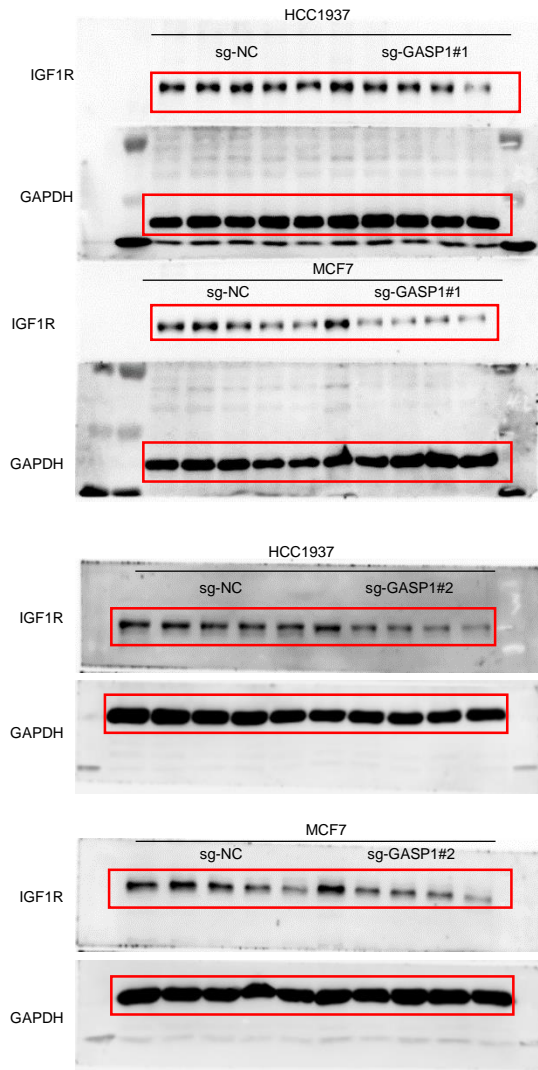**b**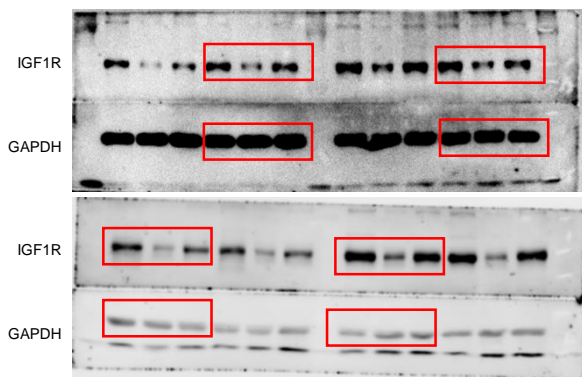**d**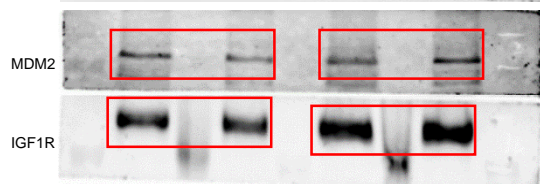**e**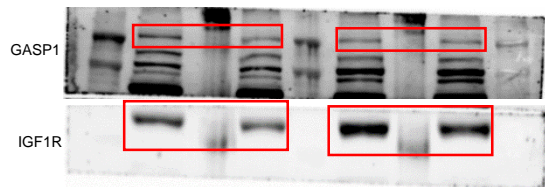**c**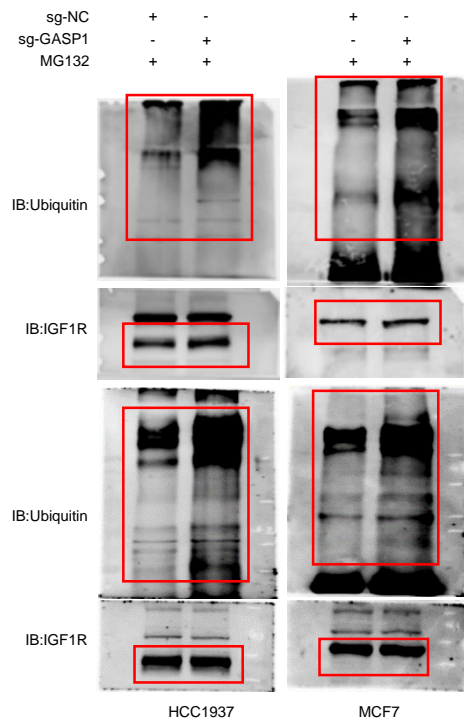**f**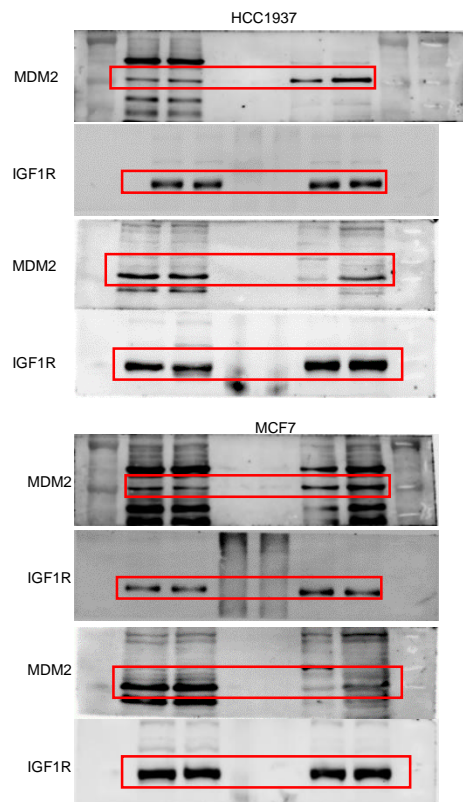

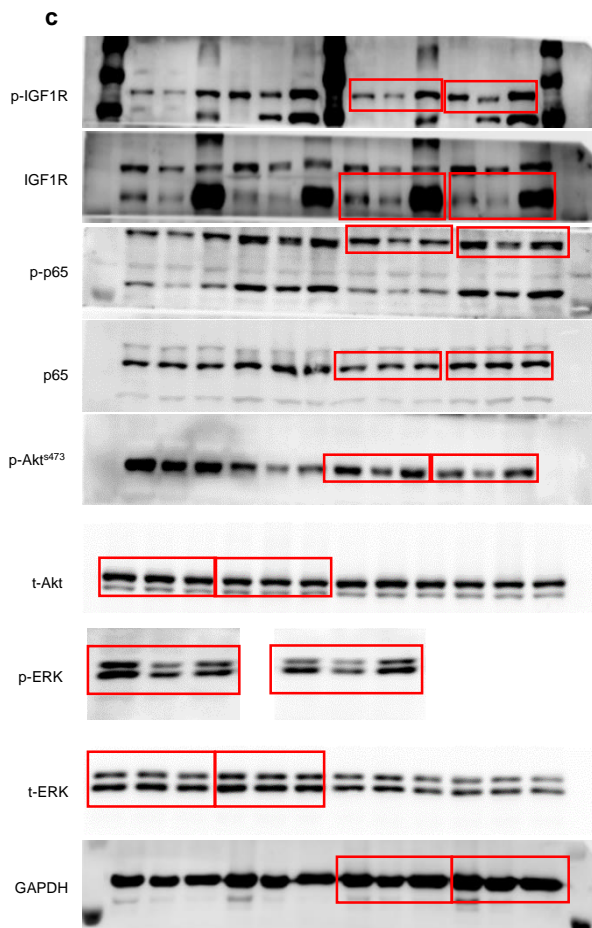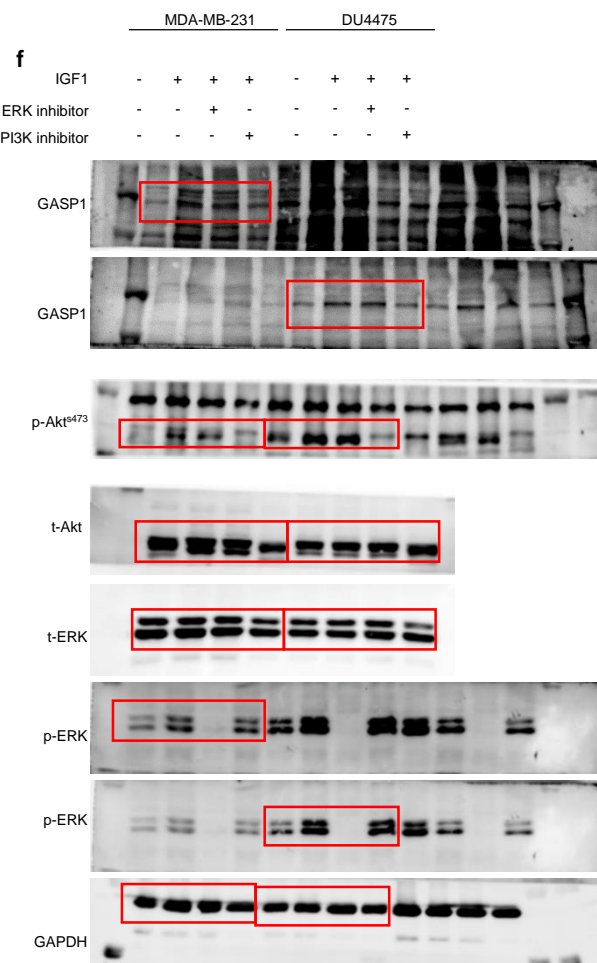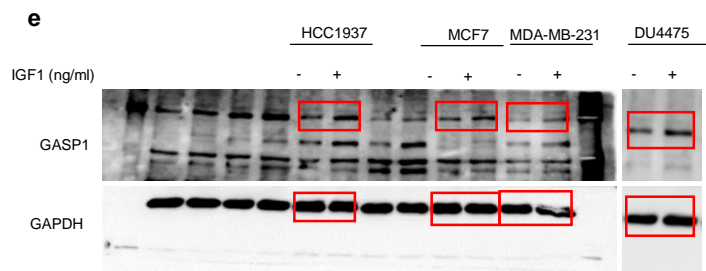

**b**

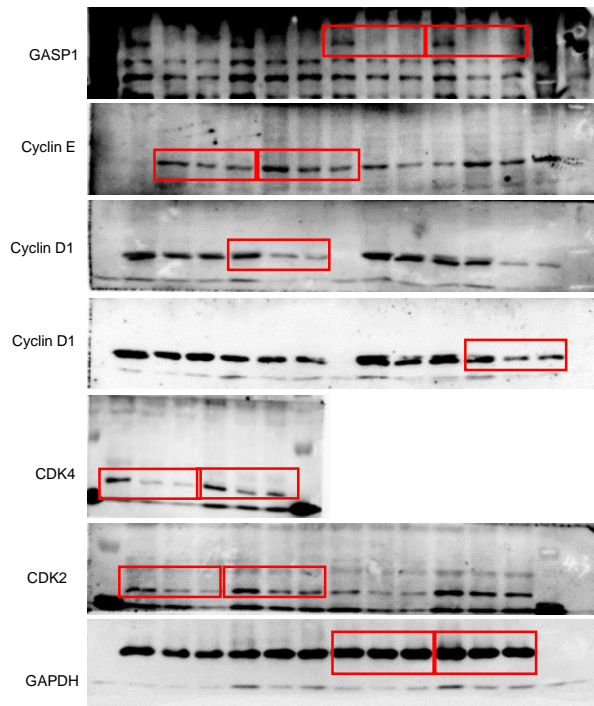

**c**

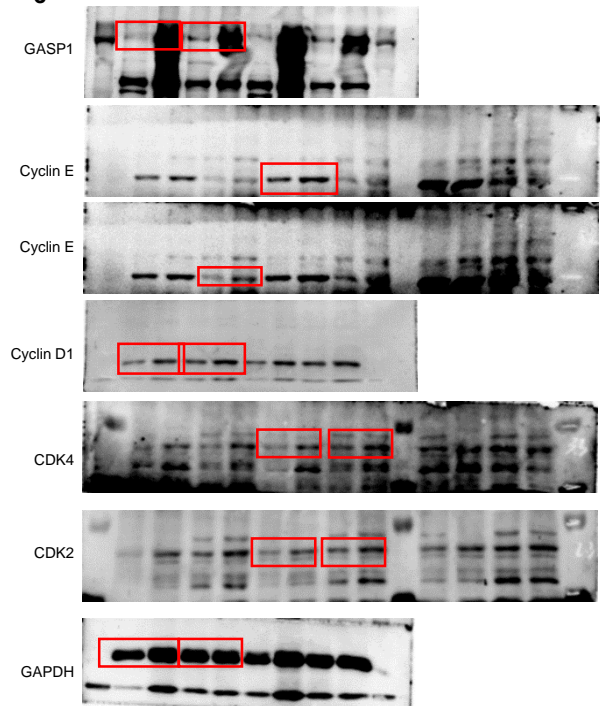



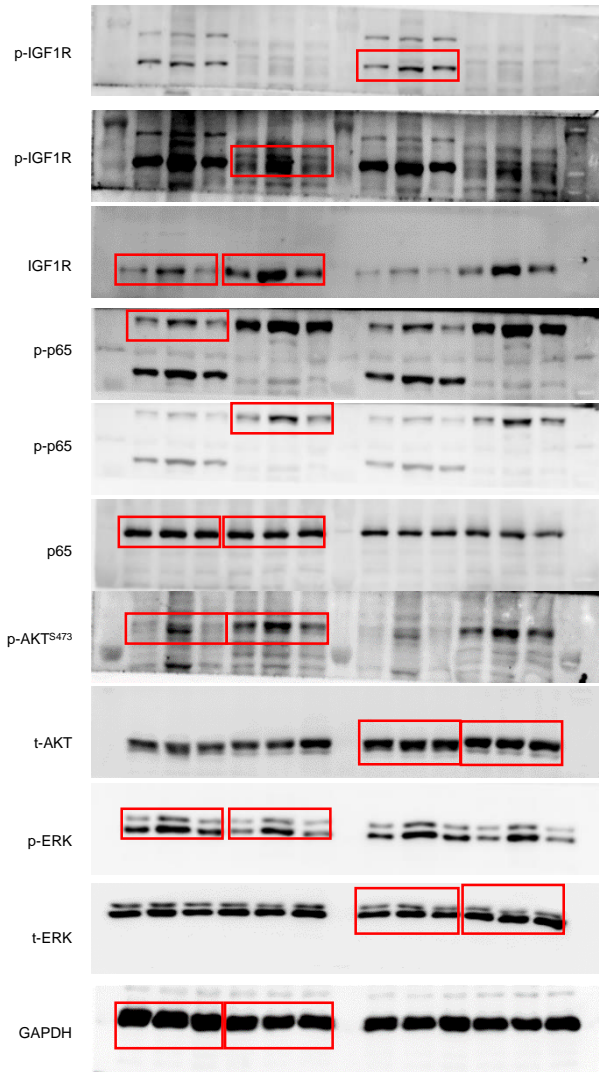

Supplement: Supplementary file 12 — Full and uncropped western blots [file 41419_2022_5198_MOESM12_ESM.pdf]
